# Regulation of flavonoids in strawberry fruits by FaMYB5/FaMYB10 dominated MYB-bHLH-WD40 ternary complexes

**DOI:** 10.3389/fpls.2023.1145670

**Published:** 2023-03-13

**Authors:** Maolan Yue, Leiyu Jiang, Nating Zhang, Lianxi Zhang, Yongqiang Liu, Yuanxiu Lin, Yunting Zhang, Ya Luo, Yong Zhang, Yan Wang, Mengyao Li, Xiaorong Wang, Qing Chen, Haoru Tang

**Affiliations:** ^1^ Country College of Horticulture, Sichuan Agricultural University, Chengdu, China; ^2^ Institute of Pomology & Olericulture, Sichuan Agricultural University, Chengdu, China

**Keywords:** anthocyanin, proanthocyanidins, strawberry, MBW complex, FaMYB5, FaMYB10

## Abstract

Anthocyanins endowing strawberry fruit red color are regulated by the MYB-bHLH-WD40 complex. By analyzing the MYBs involved in the flavonoid biosynthesis in strawberry, we found that R2R3-FaMYB5 promoted the content of anthocyanin and proanthocyanidins in strawberry fruits. Yeast two-hybrid and BiFC assays confirmed that MBW complexes connected with flavonoid metabolism were FaMYB5/FaMYB10-FaEGL3 (bHLH)-FaLWD1/FaLWD1-like (WD40). Transient overexpression and qRT-PCR analysis revealed that disparate MBW models hold different patterns in the regulation of flavonoid biosynthesis in strawberry fruits. Compared with FaMYB10, FaMYB5 and its dominant complexes showed a more specific regulatory range on strawberry flavonoid biosynthetic pathway, while FaMYB10 was more extensive. In addition, the complexes involved in FaMYB5 facilitated PAs accumulation primarily through the LAR tributary while FaMYB10 mainly by the ANR branch. FaMYB9 and FaMYB11 tremendously elicited the accumulation of proanthocyanidins by up-regulating the expression levels of both *LAR* and *ANR*, and also affected anthocyanin metabolism by changing the ratio of Cy3G and Pg3G which were constituted as two major anthocyanin monomers in strawberries. Our study also illustrated that FaMYB5-FaEGL3-FaLWD1-like directly targeted the promoters of *F3′H*, *LAR*, and *AHA10* thus committing to flavonoid accumulation. These results allow the specific members involved in the MBW complex to be deciphered and provide new insights into the regulatory mechanisms of anthocyanins and proanthocyanidins regulated by the MBW complex.

## Introduction

Cultivated strawberry (*Fragaria* × *ananassa* Duch.) is one of the world’s major fruit crops, recognized for its nutritional value and attractive appearance (Afrin et al., 2016). The edible part of a strawberry is developed from the receptacle with a large number of flavonoids. Ripe strawberry fruit mainly contains pelargonidin 3-glucoside (Pg3G) and cyanidin 3-glucoside (Cy3G) appearing bright and dark red color respectively (Da Silva et al., 2007), while in unripe fruit proanthocyanidins (PAs, also named condensed tannins) formed by the monomers or polymers of catechins and epicatechins are the mainstay (Paolocci et al., 2011). There are many factors that interfere with flavonoids metabolism, in addition to external factors such as light, temperature, and exogenous hormones, biological internal factors also affect the synthesis of flavonoids (Kadomura-Ishikawa et al., 2015; Medina-Puche et al., 2016; Balasooriya et al., 2019; Warner et al., 2021).

Flavonoids metabolic pathways have been clearly studied in model plants. The biosynthesis of anthocyanins and PAs share a common phenylpropanoid pathway, starting with phenylalanine precursor, and is catalyzed by multiple enzymes to form unstable leucoanthocyanins, then modified by glycosylation, acetylation, or methylation to generate monomeric anthocyanin substances. Leucoanthocyanins can also be catalyzed by leucoanthocyanidin reductase (LAR) and anthocyanidin reductase (ANR) to produce catechins and epicatechins, thereby entering the PAs synthesis branch (Jaakola, 2013; Cheng et al., 2015; Suvanto et al., 2020). The accumulation of flavonoids is the result of the expression of structural genes by overall orchestration. In the flavonoids biosynthesis pathway, *flavonoid 3-hydroxylase* (*F3H*) and its upstream structural genes including *chalcone synthase* (*CHS)*, *chalcone isomerase* (*CHI*) are classified as early biosynthesis genes (EBGs) which are mainly regulated by MYBs. Nevertheless, the downstream structural genes composed of *flavonoid 3′-hydroxylase* (*F3’H*), *flavonoid 3’,5’-hydroxylase* (*F3’5’H*), *dihydroflavonol-4-reductase* (*DFR*), *anthocyanidin synthase* (*ANS*), *flavonoid 3-O-glycosyltransferase* (*F3GT*), *LAR*, *ANR* go for late biosynthesis genes (LBGs) and regulated by MYB-bHLH-WD40 (MBW) ternary complex in a tissue or specific manner (Petroni and Tonelli, 2011; Xu et al., 2015). According to the number of R motifs contained in the N-termini of MYB, MYB transcription factors (TFs) are divided into four subfamilies, R1-MYB, R2R3-MYB, R1R2R3-MYB, and 4R-MYB. The specificity of distinct MBW complexes determined at least in part by MYB, and the MYB involved in the regulation of flavonoids biosynthesis are mainly R2R3-MYBs usually contain an [DE]Lx2[RK]x3Lx6Lx3R motif in its R3 domain, which is responsible for the interaction with the bHLH TF (Dubos et al., 2010).

*Fragaria vesca* has 187 MYBs or MYB-related TFs, including 23 MYBs that govern the regulation of flavonoids (Shulaev et al., 2011). Recent studies reveal that FaMYB1, FaMYB5, FaMYB9, FaMYB10, and FaMYB11 are responsible for strawberry flavonoids biosynthesis by activating or repressing structural genes (Salvatierra et al., 2013; Kadomura-Ishikawa et al., 2015; Wang et al., 2020). FvMYB3, FvMYB9, FvMYB11, FvMYB22, FvMYB64, and FvMYB105 are aligned with PAs accumulation, FvMYB10 and FvMYB41 associated with anthocyanins accumulation (Xu et al., 2021). It is thought that FaMYB1 and FcMYB1 have the core sequence (pdLNLD/EL) in the EAR motif which makes it possible to act as an active transcriptional repressor like AtMYB4 (Aharoni et al., 2001; Paolocci et al., 2011). AtMYB5 is connected with tannin biosynthesis, seed coat development, and trichome morphogenesis (Gonzalez et al., 2009; Li et al., 2009; Dubos et al., 2010); VvMYB5b activates some genes in the flavonoids pathway, while VvMYB5a has a more limited impact on the anthocyanin pathway (Deluc et al., 2006; Cavallini et al., 2014), VvMYC1 (bHLH) physically interacts with VvMYB5a, VvMYB5b to induce promoter activities of anthocyanin and/or PAs biosynthesis (Hichri et al., 2010). In 2013, Schaart filtered out an R3-FaMYB5 with a 70 aa deletion leading to a loss of the R2 domain, it is speculated that R3-FaMYB5 may repress PAs or anthocyanins (Schaart et al., 2013). In *Arabidopsis*, four TTG1-dependent regulatory complexes (TT2-TT8-TTG1, MYB5-TT8-TTG1, TT2-EGL3-TTG1, and TT2-GL3-TTG1) participate in PAs accumulation through different target genes, AtMYB5 is partially redundant with TT2 in this process (Gonzalez et al., 2009; Xu et al., 2014).In *petunia*, the specificity of MBW complex determined by AN2/PH4 (MYB)-AN1 (bHLH)-AN11 (WD40) is of benefit to anthocyanin biosynthesis in petals, while AN4 (MYB)-AN1-AN11 plays a role in the coloration of flower tube and anther, PH4 can also interact with JAF13 (bHLH) in the petal epidermis, drive anthocyanin biosynthesis (Quattrocchio et al., 2006; Verweij et al., 2016). For Apple (*Malus× domestica*), *MdMYBA*, *MdMYB1*, and its allele *MdMYB10* are the main determinant of cultivar fruit color differences (Takos et al., 2006; Ban et al., 2007; Chagne et al., 2013). It has been reported that efficient induction of anthocyanin biosynthesis by MdMYB10 depends on the co-expression of MdbHLH3 and MdbHLH33, whereas, in some species (strawberry, pear, peach, and rose), MYB10 TFs perform poorly with bHLH3 (Lin-Wang et al., 2010; Xie et al., 2012). In strawberries, FaMYB10 is also considered to be involved in the formation of the MBW complex, and speculate that the MBW complex model that regulates anthocyanin metabolism is FaMYB10-FaEGL3-FaTTG1(WD40), in which FaMYB1 competitively bind to FaEGL3 to balance anthocyanin accumulation (Schaart et al., 2013), while some believe that the bHLH combined is FabHLH3 or 33 (Wei et al., 2018).

Anthocyanin needs to be transported from the cytosol to the vacuole for stable storage, procyanidin monomers require to be transported to the vacuole to polymerize into PAs likewise. Three main types of transporters have been reported: multidrug and toxic compound extrusion (MATE), glutathione-S-transferase (GST), and H^+^-ATP (autoinhibited H^+^-ATPase isoform l0, AHA10) (Zhao et al., 2010).In *Arabidopsis*, *TT19* (*GST*) mutations affect both anthocyanin accumulation in vegetative tissues and PAs accumulation in the seed coat, while its homologous gene *PpGST1* is critical for anthocyanin transport but not PAs and its expression was stimulated by PpMYB10.1 (Zhao et al., 2020). AtTT12 (MATE) localizes to the tonoplast and can transport Cy3G (Grotewold and Davies, 2008; Sun et al., 2012), FaTT12 (MATE) is specifically involved in PAs accumulation in strawberry fruits (Chen et al., 2018). AtAHA10 (H^+^-ATPase) is responsible for seed coat PAs production (Zhao et al., 2010). In the process of fruit development, the main regulators of flavonoids biosynthesis are MYB TFs. In recent years, research on the regulation of flavonoids biosynthesis has tended to focus on MYB, bHLH and WD40, and has been carried out on many Rosaceae crops, including strawberry, and made great achievements. Up to now, the MBW complex relevant to flavonoids metabolism in strawberry revolving around FaMYB9/10/11. It has commonly been assumed that MYB10 is thought to play a central role in anthocyanin metabolism, and FaMYB9/11 are closely related to PA metabolism. However, the MBW complex based on MYB10 is still controversial, and the exact members involved are indetermination. Previously published studies on *Arabidopsis* and *petunia* imply that disparate MBW regulatory models mediated flavonoid metabolism in a tissue-specific manner (Xu et al., 2014; Verweij et al., 2016). However, the multiple MBW ternary complexes and its target genes at different developmental stages of strawberry fruit are still indetermination.

In previous study, we used comparative transcriptomics and metabolomics to evaluate the effects of FaMYB5 on strawberry flavonoid and flavonoid-related pathway, further explored the regulation of R2R3-FaMYB5 *via* lncRNAs and FaBT2 at the transcription and protein levels (Jiang et al., 2023). Herein, in order to get the full landscape of the MBW complex in strawberry, we took advantage of molecular methods to screen the interacting members that may form the MBW complex and regulate the flavonoids in strawberry fruits. Using agrobacterium transient transformation, High performance Liquid Chromatography (HPLC), and qRT-PCR strategies to appraise the impact of MBW complexes FaMYB5/10-FaEGL3-FaLWD1/FaLWD1-like on the biosynthesis of strawberry fruit flavonoids, and further confirmed that FaMYB5-FaEGL3-FaLWD1-like stimulate the accumulation of anthocyanins and PAs by directly active the activity of *F3′H*, *AHA10, LAR* promoters. And also revealed that the MBW complexes involving FaMYB9/11 mainly regulate the biosynthesis of PAs through *LAR* and *ANR*. This study has identified the specific MBW complexes that regulate anthocyanin and PAs biosynthesis in strawberry fruits and explored the intrinsic molecular mechanism. Provide a theoretical basis for improving strawberry fruit coloring in production practice from a molecular perspective.

## Materials and methods

### Plant material and sample collection

Red strawberry (*Fragaria × ananassa*) ‘Benihoppe’ and its natural white-fleshed mutant ‘Xiaobai’ were grown in a greenhouse at Sichuan agricultural university, China. Six fruit ripening stages defined as small green (SG), big green (BG), white (W), initial red (IN), partial red (PR), and full red (R) were harvested in December (Yue et al., 2022). The skin (outer red layer including achenes) and flesh (inner layer) were manually separated, then frozen in liquid nitrogen immediately.

### RNA extraction and qRT-PCR analysis

The total RNA of fruit samples was extracted using the improved CTAB method (Chen et al., 2012), and then reversed by PrimeScript™ RT reagent Kit with gDNA Eraser (TaKaRa, Dalian). The cDNA was diluted quadruple and used as the template for gene cloning and qRT-PCR analysis. qRT-PCR was performed by a CFX Connect real-time system (CFX, Bio-Rad, USA) and the interspacer 26S-18S RNA was used as reference gene (Wei et al., 2017). Primers were designed by Beacon designer 8 software (**Supporting Information Table S1**) and the relative expression level was analyzed using 2^-ΔΔCt^ method (Livak and Schmittgen, 2001). The amplification program was as follows: one cycle of 30 s at 95°C, followed by 40 cycles of 5 s at 95°C, 30 s at 55°C and 30 s at 72°C. The system without cDNA template was used as a negative control. Three biological replicates were set, and each biological replicate was set with three technical replicates.

### Screening for candidate genes related to flavonoids

Candidate genes were obtained by homologous alignment in strawberry according to the MBW members involved in flavonoids metabolism reported in model plants *Arabidopsis*, *Petunia* and strawberry. Primers were designed based on the CDs sequences of these genes (**Supporting Information Table S1**), and PCR amplification was carried out with the ‘Benihoppe’ cDNA as the template.

The accession numbers of genes and promoters in this study can be found in Genebank: *FaMYB1* (MW700312), *R3-FaMYB5* (OM948808), *R2R3-FaMYB5* (MW700311), *FaMYB10* (OK001452), *FaEGL3* (MW700313), *FabHLH33B* (OM948809), *FabHLH33* (ON227181), *FaGL3* (OM948810), *FaMYC1* (OM948806), *FabHLH3* (OM937882), *FaTTG1* (OM948807), *FaLWD1* (MW700314), *FaLWD1-like* (MW700315), *FaMYB44-like* (ON227180), *F3’H* promoter (OK001460**)**, *LAR* promoter (OK001458), *AHA10* promoter (ON246274), *TT12* promoter (ON246275).

### Anthocyanins and PAs determination

Anthocyanins content was determined using HPLC methods. 0.2 g frozen strawberry fruit (FW) samples were ground and extracted in 2 mL extraction solution (1% HCL-containing methanol solution) for 48 h at 4°C in darkness, and collect the supernatant after centrifugation at 13,000 r/min for 15 min. Add 2 mL extraction to the sample residue, repeat the extraction (for 24 h), collect the supernatant, and mix with the first extraction. Filtered the crude extract with a 0.45 µm Nylon filterer and injected into brown HPLC sample bottles. A 250 mm × 4.6 mm i.d, 5 µm reversed-phase Silgreen ODS C18 column (Greenherbs science and technology, Beijing, China) was used to separate and determine anthocyanins. Mobile phase consists of 5% formic acid (A), and methanol (B). Linear gradient (95-0%) of formic acid in methanol was used for 20 min, followed by 100% methanol for 5 min. The column temperature was kept at 25°C, the flow rate was 1 ml/min, and chromatograms were recorded at 510 nm. Three biological replicates were set, and each biological replicate was set with three technical replicates.

PAs were determined by DMAC (4-dimethylaminocinnamaldehyde) method. 1.5 g frozen strawberry fruits were grinded with liquid nitrogen and resuspended in 20 ml PAs extract (acetone: water: acetic acid = 150 ml: 49 ml: 1 ml). The mixture was shaken at 150 r/min for 1 h, then centrifuged for 20 min at 10,000 × g, carefully aspirate the supernatant and analyzed at 640 nm in a 96-well microplate reader (Thermo Fisher Scientific, Waltham, MA, USA).

### Transient expression in strawberry fruits

Full-length CDS sequences of MYBs, bHLHs, and WD40s were amplified from the cultivar ‘Benihoppe’, and fusion into the modified pCAMBIA1301 vector with enhanced CaMV35S promoter through homologous recombination method. Agrobacterium solution was transferred into ‘Benihoppe’ and ‘Xiaobai’ white stage fruits *via* agrobacterium-mediated transformation method, and the steps of transfection were described in detail elsewhere (Lin et al., 2018).

### Yeast two-hybrid study

The principle of the Yeast two-hybrid study was followed by Matchmaker™ Gold Yeast Two-Hybrid System User Manual. Bait proteins were expressed as a fusion to the Gal4 DNA-binding domain (pGBKT7), while prey proteins were expressed as fusions to the Gal4 activation domain (pGADT7). The controls were as follows, positive control: Y2HGold [pGBKT7-53] and Y187 [pGADT7-T]; negative control: Y2HGold [pGBKT7-Lam] and Y187 [pGADT7-T].

### Bimolecular fluorescent complimentary assay

Two target genes were fused into the vector modified from pSAT1-nEYFP-C1 and pSAT1-cEYFP-N1 (Citovsky et al., 2006). The constructed vectors were transformed to Agrobacterium GV3101, cultured overnight at 28°C in YEP medium supplemented with appropriate antibiotics, then centrifuged and resuspended the bacteria in MMA solution (10 mM MES, 10 mM MgCl_2_, 500 μM acetosyringone) and incubated at room temperature for 3 h on a shaker. The bacteria was centrifuged and resuspended in MMA solution to a final OD600 of 1.0. Next, use a plastic syringe to inject the back of the tobacco vane. After agroinfiltration, the treated plants were maintained in the greenhouse for 3 days before observation under a confocal microscope (Olympus FV1000).

### Dual-luciferase assay

The promoter regions of *F3′H*, *LAR*, *TT12*, and *AHA10*, 1.5-2 kb upstream from the translation start site (TSS) were amplified and combined into the pGreenII0800-LUC vector as reporters. Genes fused into pCAMBIA1301-d35SN were used as effectors and the empty pCAMBIA1301-d35SN was as a negative control. The preparation of bacterial solution was in accordance with the BiFC method mentioned above. Dual-luciferase assay was performed as described previously (Liu et al., 2008). Fire luciferase (LUC) and Renilla luciferase (REN) were detected using the Dual-Luciferase Reporter Assay System kit (Promega, USA).

## Results

### Bioinformatic analysis of 15 identified MBW members in octoploid strawberries

Based on the reported MBW composition in typical model plants and horticultural species for studying flavonoids metabolisms, such as *Arabidopsis*, *petunia hybrida*, *maize*, *Malus × domestica*, and *Vitis vinifera*, we sorted out the MBW members in octoploid strawberries by bioinformatic analysis. Finally, a total of 15 candidate MBW members were selected and amplified from cultivated strawberries, including six MYB TFs (FaMYB1/9/10/11, R3-FaMYB5, and R2R3-FaMYB5), six bHLH TFs (FaEGL3, FabHLH33/33B/3, FaMYC1, and FaGL3) and three WD40 TFs (FaTTG1, FaLWD1, and FaLWD1-like).

**Figure 1:**
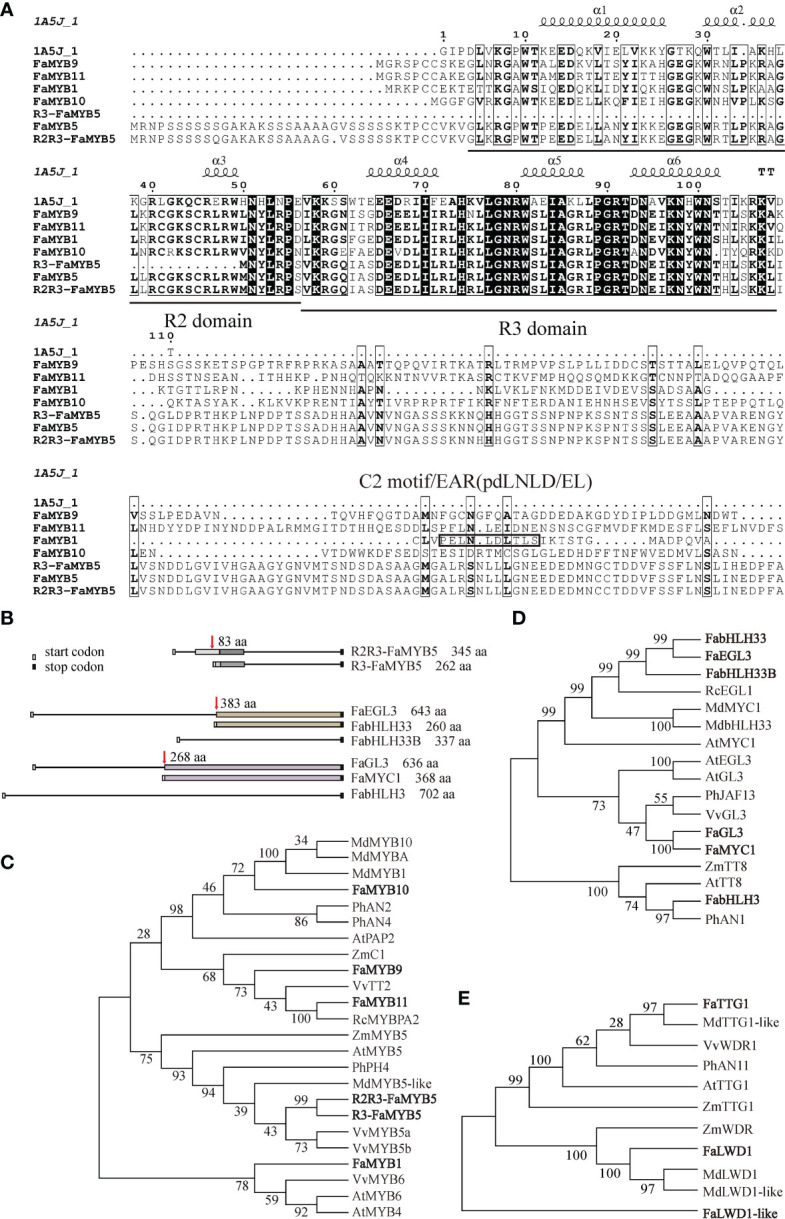
Bioinformatic analysis of 15 identified strawberry transcriptional regulators. **(A)** Multiple protein sequence alignment of strawberry MYB transcription factors. Reference sequence of 1A5J_1 download from https://www.rcsb.org/structure/1a5j. **(B)** Sequence model diagrams for different MYBs and bHLHs. The same color box represents the exact same amino acid sequence. **(C-E)** Phylogenetic analysis of selected MYBs, bHLHs and WD40s (bold font) with their homologous proteins from other species. The phylogenetic tree was constructed by the Neighbor-Joining Tree Method in MEGA6, and the bootstrap was 1,000 replicates. MdMYB10 (ABB84753), MdMYBA (BAF80582), MdMYB1 (ABK58136), PhAN2 (BAP28593.1), PhAN4 (ADQ00392.1), AtPAP2 (OAP17920.1), ZmC1 (ACG27887.1), VvTT2 (RVW43997.1), RcMYBPA2 (QBQ01525.1), ZmMYB5 (XP_008656780.1), AtMYB5 (NP_187963.1), FaMYB5 (XP_004295005.1), PhPH4 (AAY51377.1), MdMYB5-like (NP_001315719.1), VvMYB5a (AAS68190.1), VvMYB5b (AAX51291.1), VvMYB6 (XP_002273328.1), AtMYB6 (NP_192684.1), AtMYB4 (NP_195574.1), RcEGL1 (XP_024176861.1), MdMYC1 (XP_028961533.1), MdbHLH33 (ABB84474.1), AtMYC1 (AEG74457.1), AtEGL3 (NP_001185302.1), AtGL3 (NP_001332705.1), PhJAF13 (AAC39455.1), VvGL3 (RVX09227.1), ZmTT8 (ONM52414.1), AtTT8 (NP_192720.2), PhAN1 (AAG25927.1), MdTTG1-like (NP_001315674.1), VvWDR1 (NP_001268101.1), PhAN11 (AAC18914.1), AtTTG1 (CAC10524.1), ZmTTG1 (NP_001310302.1), ZmWDR (NP_001169326.1), MdLWD1 (XP_008357691.1), and MdLWD1-like (XP_008378323.1). Md, *Malus× domestica*; Fa, *Fragaria × ananassa*; Vv, *Vitis vinifera*; Ph, *Petunia* × *hybrid*; At, *Arabidopsis thaliana*; Zm, *Zea mays*; Rc, *Rosa chinensis*.

As previously reported, R2R3-FaMYB5 had an intact R2 domain compared with the former investigated R3-FaMYB5, another start codon can be found at 83 aa before R3-FaMYB5, which could encode an MYB5 protein with a complete R2R3 domain (**Figures 1A, B**; Schaart et al., 2013; Jiang et al., 2023). Sequence alignment results indicated that FaMYB1, R2R3-FaMYB5, FaMYB9, FaMYB10, and FaMYB11 belong to R2R3-type MYB TFs, in which FaMYB1 had an EAR repression motif (pdLNLD/EL) of flavonoids on the C2 domain (**Figure 1A**; Aharoni et al., 2001). The phylogenetic analysis reflected that the repressor FaMYB1 was clustered into a single clade, while R2R3-FaMYB5, R3-FaMYB5, FaMYB9, FaMYB11, and FaMYB10 were clustered into another clade with different branches. R2R3-FaMYB5 and R3-FaMYB5 were most similar to the grape VvMYB5a/5b, whereas FaMYB9/11 shared a close similarity with VvTT2 (**Figure 1C**). Among the six bHLH TFs, FaEGL3 encoded a 643 aa protein with a close phylogenetic relationship to RcEGL1 (*Rosa chinensis*), and a translation start codon at 383 aa in FaEGL3 would lead to FabHLH33, the similarity between FabHLH33 and FabHLH33B (identity = 75.07%) was the highest. Similarly, after 268 aa in FaGL3, a shorter FaMYC1 with a length of 368 aa was encoded. FabHLH3 (702 aa) was the longest of the six candidate bHLH TFs, and had low sequence similarities to the other five bHLH TFs (**Figures 1B, D**). In addition, three WD40 TFs were picked out by homologous alignment with PhAN11 and AtTTG1, the sequence similarity between FaTTG1 and PhAN11 was as high as 82%, FaLWD1 and FaLWD1-like also shared more than 50% sequence similarity with PhAN11. FaTTG1, FaLWD1, and FaLWD1-like were clustered into independent clades, and FaTTG1 was most similar to apple MdTTG1-like (**Figure 1E**).

### Effects of candidate TFs on anthocyanins and PAs in strawberry fruits

**Figure 2:**
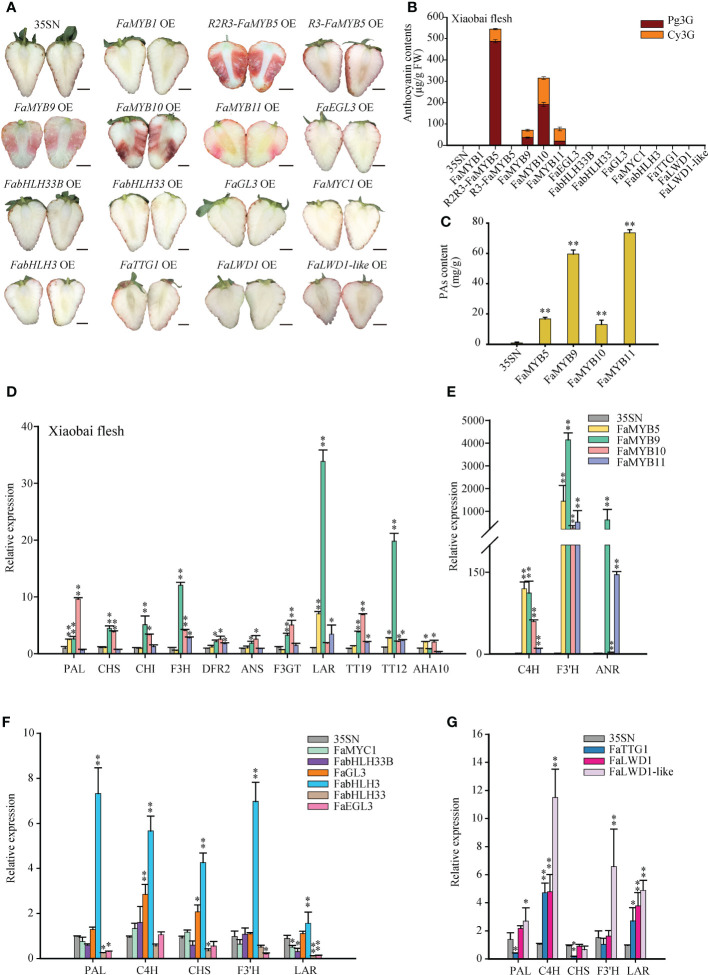
Effects of transient overexpression 15 TFs on anthocyanins and PAs in strawberry fruits. **(A)** The phenotype of transient overexpression 15 TFs in ‘Xiaobai’ fruits. OE, overexpressed sample; 35SN (empty vector) was as control, scale bars represent 10 mm. **(B)** Analysis of Cy3G and Pg3G by HPLC in ‘Xiaobai’ flesh. **(C)** The content of PAs was determined by the DMAC method. **(D-G)** QRT-PCR analysis of relative expression levels of key structural genes and transport factors in the flavonoid biosynthesis pathway. Error bars are SEs for three replicates, statistical significance was determined by Student’s t-test (**, *P* < 0.01; *, *P* < 0.05).

To ascertain the effects of these 15 TFs on anthocyanins and PAs, a transient overexpression (OE) strategy was implemented on ‘Benihoppe’ and ‘Xiaobai’ fruits. Compared with 35SN, *R2R3-FaMYB5* OE, *FaMYB9/10/11* OE reproducibly restored pigments accumulation in fruits, particularly in *R2R3-FaMYB5* OE and *FaMYB10* OE, while *FaMYB9* OE or *FaMYB11* OE arose a faint and diffuse pigmentation (**Figure 2A**; **Supplementary Figure S1**). HPLC analysis in the flesh of 35SN samples suggested that Cy3G and Pg3G were not detected ‘Xiaobai’, while no Cy3G was found in ‘Benihoppe’. However, *R2R3-FaMYB5*, *FaMYB9*, *FaMYB10*, and *FaMYB11* could promote the accumulation of Cy3G and Pg3G in ‘Xiaobai’ flesh, restored Cy3G in ‘Benihoppe’ flesh (**Figure 2B**; **Supplementary Figure S2**). Ripe strawberry fruits mainly contained bright red Pg3G and dark red Cy3G. In *R2R3-FaMYB5* OE, Cy3G accounted for 10.4% of the total anthocyanin content, while the proportion stood at 39.1% in *FaMYB10* OE, resulting the *R2R3-FaMYB5* OE flesh appeared bright red and *FaMYB10* OE flesh dark red. And the total anthocyanin content of *FaMYB5* OE was higher than that of *FaMYB10* OE in both ‘Xiaobai’ and ‘Benihoppe’ flesh. Anthocyanins are weakly affected by *R3-FaMYB5*, and there was no further delving into *R3-FaMYB5* followed (the *MYB5* mentioned below without special comment referred to the *R2R3-FaMYB5*). In the overexpressed samples of other genes could not detect any pigment. Interestingly, the content of PAs in *FaMYB5/9/10/11* OE was significantly increased, especially in *FaMYB9/11* OE (**Figure 2C**).

qRT-PCR analysis showed that the mRNA levels of *PAL*, *C4H*, *F3′H*, *LAR*, *TT12*, and *AHA10* increased significantly in *FaMYB5* OE, especially *C4H* and *F3′H*. In *FaMYB10* OE, the vast majority of detected gene expressions are more or less up-regulated except *LAR*, it was worth noting that *TT19* was extremely significantly up-regulated among the three transport factors. In *FaMYB9* OE, the set of genes (except *AHA10*) were all enhanced, especially the PAs branch directed genes *F3′H*, *LAR*, *ANR*, and PAs transporter *TT12* (Chen et al., 2018). *C4H*, *F3′H*, *LAR*, *ANR*, *TT19*, *TT12* were significantly up-regulated in *FaMYB11* OE (**Figures 2D, E**). The HPLC and qRT-PCR results of *FaMYB9* OE and *FaMYB11* OE demonstrated that those two *MYBs* not only increased the accumulation of anthocyanins by adjusting the ratio of Cy3G to Pg3G, but were also responsible for the synthesis of PAs in strawberry fruits. In *bHLHs* and *WDs* OE, the magnitude of the upregulated genes was not as strong as that in *MYBs* OE (**Figures 2F, G**). The expression levels of the above TFs were shown in **Supplementary Figure S3**.

### Potential MBW ternary complexes were identified

**Figure 3:**
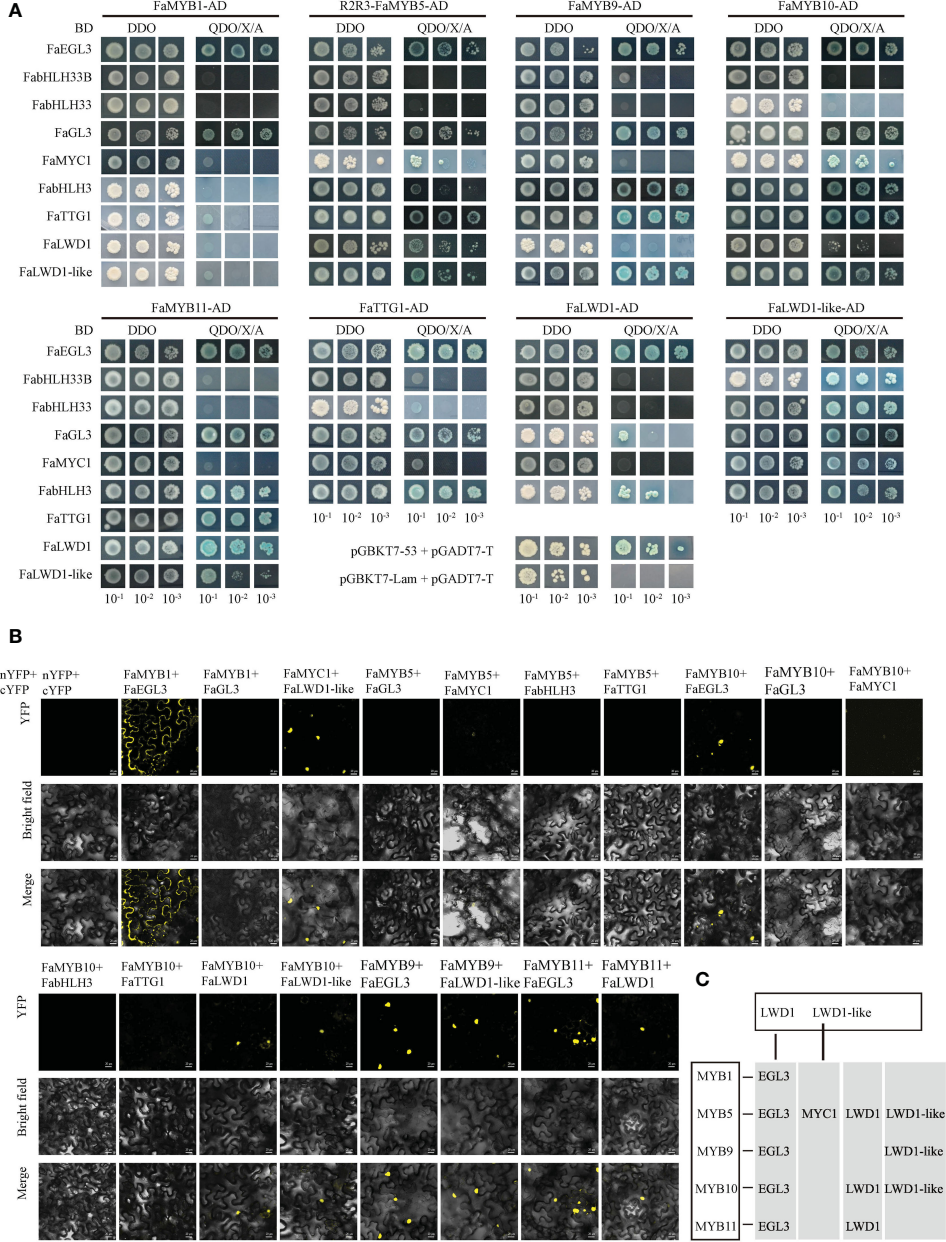
Screening of interacting proteins. **(A)** Validation of interacting proteins using the Y2H method. DDO: SD/-Trp/-Leu, QDO/X/A: SD/-Trp/-Leu/-His/-Ade/+X-α-gal/+AbA, positive control was pGADT7-T + pGBKT7-53 and pGADT7-T + pGBKT7-Lam as negative control. **(B)** Protein-protein interaction verified by BiFC assay. Scale bars represent 20 µm. **(C)** Diagram of the interaction relationship confirmed by BiFC and Y2H.

Y2H and BiFC assays were performed to verify the interaction between MYBs, bHLHs, and WD40s. The Y2H results showed that FaMYB1/5/9/10/11 interacted with FaEGL3 or FaGL3 among the six bHLH TFs, and FaMYB5/10 could cooperate with FaMYC1, FaMYB9/10/11 interacted with FabHLH3. While, no significant interactions were observed between these MYBs and FabHLH33B/33. In addition, there were also protein interactions between FaMYB5/9/10/11 and FaTTG1/FaLWD1-like, and weaker interactions also existed between FaMYB5/10 and FaLWD1.The three WD40 TFs, FaTTG1, FaLWD1, and FaLWD1-like, all cooperated with FaEGL3/FaGL3/FabHLH3, as well as partially interacted with FabHLH33B/FabHLH33/FaMYC1 (**Figure 3A**). The positive interactions screened using the Y2H method were subsequently confirmed by BiFC assay. The result demonstrated that FaMYB1 did not interact with any WDs, so there was no further discussion below. FaMYB9 interacted with FaEGL3/FaLWD1-like, while FaMYB11 interacted with FaEGL3/FaLWD1. Surprisingly, no fluorescent signals were detected between FabHLH3/FaGL3/FaTTG1 and FaMYB5/10. The signals for FaMYB5/10 and FaMYC1 proteins were faint, while other positive interactions could still be observed by BiFC (**Figure 3B**; Jiang et al., 2023). Therefore, we mainly explored the consistent interactions selected by Y2H and BiFC in the following experiments, especially the FaMYB5/10, FaEGL3, and FaLWD1/FaLWD1-like TFs that had the potential ability to form the MBW complex (**Figure 3C**).

### The complexes involving FaMYB5/10 promoted the accumulation of anthocyanins and PAs in strawberry fruits

**Figure 4:**
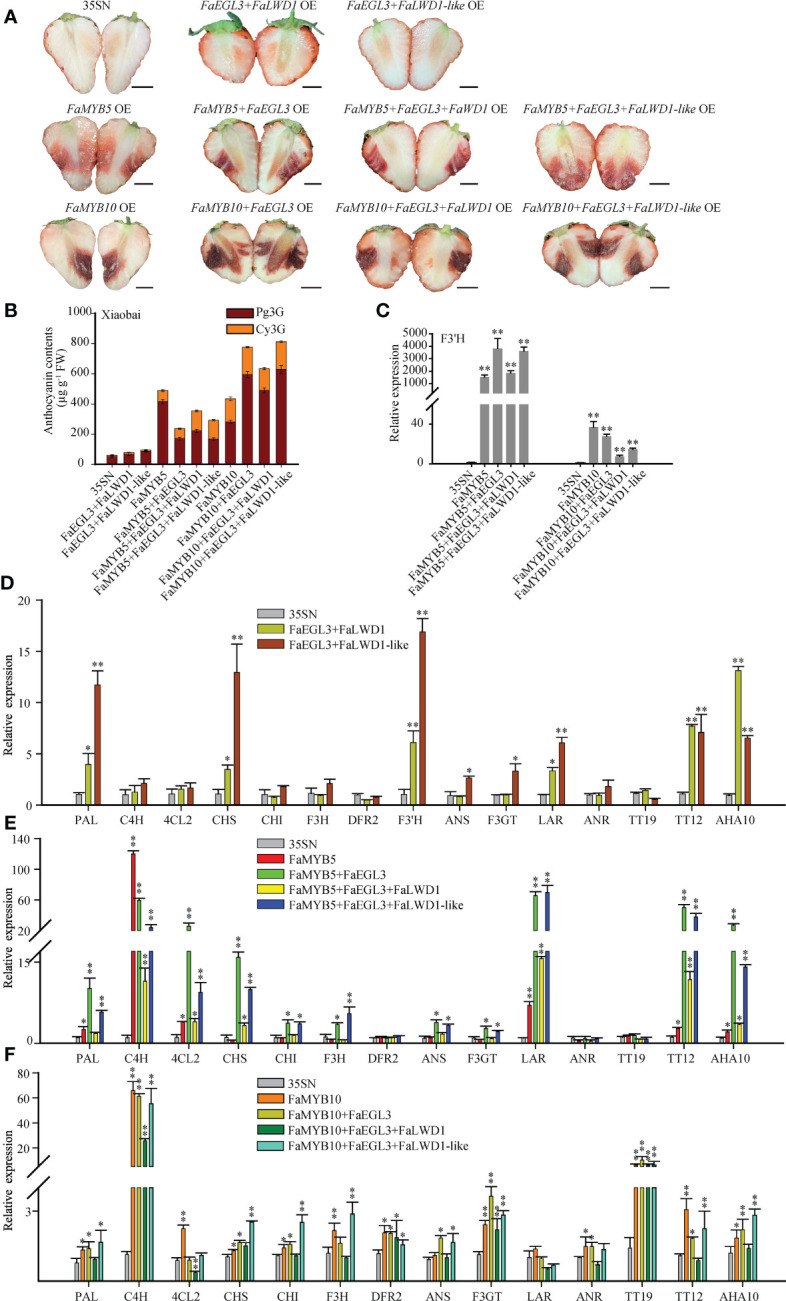
Effects of FaMYB5/10 dominated MBW complexes on flavonoid biosynthesis in strawberries. **(A)** The phenotype of co-overexpressed TFs in ‘Xiaobai’. 35SN (empty vector) was as control. Scale bars represent 10 mm. **(B)** Analysis of Cy3G and Pg3G by HPLC in ‘Xiaobai’. **(C-F)** qRT-PCR analysis of relative expression levels of key structural genes and transport factors in the flavonoid biosynthesis pathway. Error bars are SEs for three replicates, statistical significance was determined by Student’s t-test (**, *P* < 0.01; *, *P* < 0.05).

To explore the molecular mechanism of putative MBW complexes involved in flavonoids metabolism, FaMYB5/10, FaEGL3, FaLWD1, and FaLWD1-like TFs were co-overexpressed in ‘Xiaobai’ fruits. As shown in **Figure 4A**, *FaEGL3*+*FaLWD1*/*FaLWD1-like* OE showed a pale reddening, while *FaMYB5*/*10*+*FaEGL3*, *FaMYB5*/*10*+*FaEGL3*+*FaLWD1*, and *FaMYB5*/*10*+*FaEGL3*+*FaLWD1-like* OE turned red violently. HPLC results illustrated that the main anthocyanins were Pg3G, followed by Cy3G in ‘Xiaobai’ OE samples. Compared with the control, anthocyanin content was slightly increased in *FaEGL3*+*FaLWD1*/*FaLWD1-like* OE, but when they were overexpressed alone, no anthocyanin could be detected (**Figure 2B**). The content of anthocyanins increased significantly when co-overexpressed with *FaMYB5*/*10*, mainly due to the substantial increase of Pg3G (**Figure 4B**).

The qRT-PCR results revealed that the relative expression levels of some structural genes and flavonoid-related transporters were up-regulated in *FaEGL3*+*FaLWD1*/*FaLWD1-like* OE, for instance: *PAL*, *CHS*, *F3’H*, *LAR*, *TT12*, and *AHA10* (**Figure 4D**). Moreover, the expression abundance of those detected genes was greatly expanded by the addition of *FaMYB5*, especially in *FaMYB5*+*FaEGL3* and *FaMYB5*+*FaEGL3*+*FaLWD1-like* OE, while *DFR2*, *ANR*, and *TT19* remain unchanged (**Figures 4C, E**). When *FaMYB10* was co-overexpressed with *FaEGL3* and *FaLWD1* or *FaLWD1-like*, the expression levels of *C4H*, *F3’H*, *F3GT*, and *TT19* were enhanced 4-fold to 60-fold compared with the control. Nevertheless, *PAL*, *CHS*, *CHI*, and *ANS* were up-regulated only when *FaMYB10* was co-expressed with the other three TFs. The relative expression levels of most structural genes in co-overexpressed samples were higher than that of *FaMYB10* OE alone. Unexpectedly, *LAR* was hardly regulated by *FaMYB10* (**Figures 4C, F**).

### MBW complexes dominated by FaMYB5 directly targeted the promoters of *F3′H*, *AHA10*, and *LAR* to regulate anthocyanins and PAs

**Figure 5:**
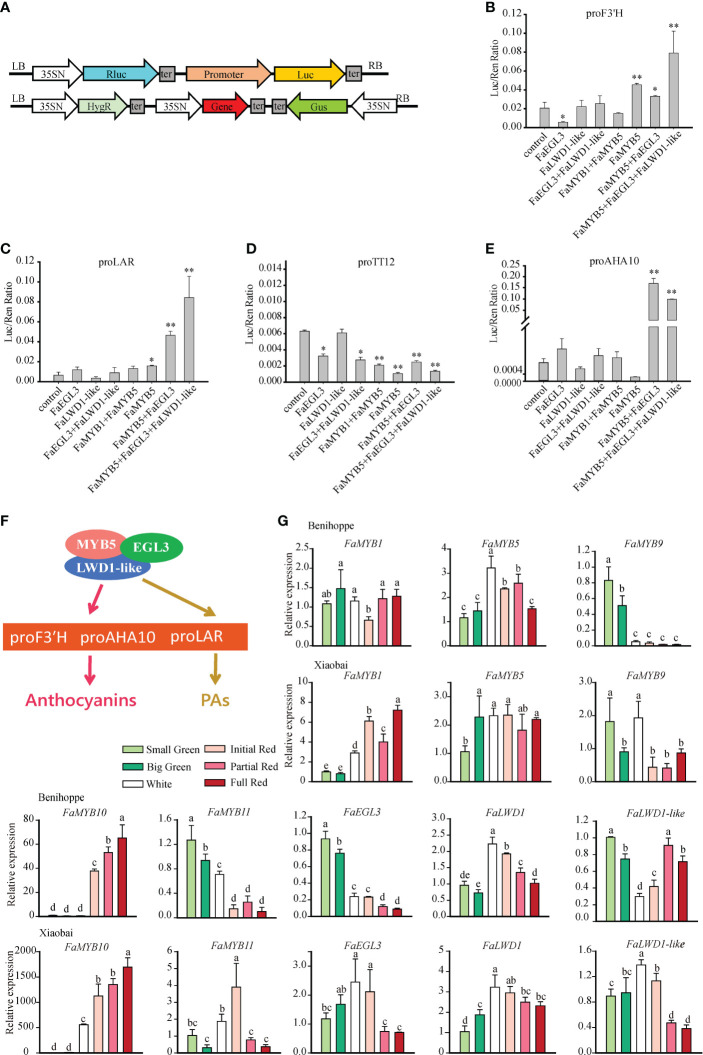
The effect of FaMYB5 and its involved MBW complex on target genes. **(A)** Schematic diagram of Dual-luciferase transient expression vectors. Promoters were combined into pGreenII0800-LUC vector as reporters, genes fused into pCAMBIA1301-d35SN were used as effectors and the empty pCAMBIA1301-d35SN was as a negative control. **(B-E)** Validation of the activation effect by FaMYB1, FaMYB5, FaEGL3, and FaLWD1-like on four promoters using dual luciferase assay in tobacco leaves. Error bars are SEs for three replicates, statistical significance was determined by Student’s t-test (**, *P* < 0.01; *, *P* < 0.05). **(F)** The regulation complex model on anthocyanins and PAs in strawberry fruit. FaMYB5/FaEGL3/FaLWD1-like MBW ternary complex regulates anthocyanins and PAs by directly binding to the promoters of F3′H, AHA10 and LAR. **(G)** Expression Patterns of the *FaMYB1, FaMYB5, FaMYB9, FaMYB10, FaMYB11, FaEGL3, FaLWD1, FaLWD1-like* in different tissues of ‘Benihoppe’ and ‘Xiaobai’. Error bars are SEs for three replicates, multiple comparisons were tested using Turkey’s test and significant differences (*P* < 0.05) are indicated by different letters.

Given that the activation ability of MBW complex on the target gene is stronger than MYB alone, we analyzed *F3′H*, *LAR*, *TT12*, and *AHA10* promoter activities by dual-luciferase assay in tobacco. The promoter was combined into the pGreenII0800-LUC vector as a reporter, gene fused into pCAMBIA1301-d35SN was used as an effector (**Figure 5A**). The results found clear support that *F3′H* and *LAR* promoters could be significantly activated by *FaMYB5* or *FaMYB5*+*FaEGL3*, and exhibited the highest activities in the combination of *FaMYB5*, *FaEGL3*, and *FaLWD1-like*. It was worth noting that the activation intensity of the *AHA10* promoter was nearly 300 times higher in *FaMYB5*+*FaEGL3* or *FaMYB5*+*FaEGL3*+*FaLWD1-like* than the control. More importantly, the activities of *F3′H*, *LAR*, and *AHA10* promoters would not be enhanced without *FaMYB5*. However, the activity of the *TT12* promoter was not advanced in any of the co-expressed combinations (**Figures 5B–E**). In summary, it was obvious that *FaMYB5*+*FaEGL3*, *FaMYB5*+*FaEGL3*+*FaLWD1-like* regulate anthocyanins and PAs by directly activating the promoter of *F3′H*, *LAR*, and *AHA10* (**Figure 5F**). The combination of the suppressor FaMYB1 and the activator FaMYB5 was also performed aiming to assess whether the negative regulator FaMYB1 affected the activation ability of the positive regulator FaMYB5. We found that FaMY5 alone significantly enhanced the activity of *F3’H* and *LAR* promoter, but the activity was weaker than that of the control after the joining of FaMYB1, while this combination had little effect on the activity of the other two promoters yet (**Figures 5B, C**).

### Expression patterns of the MBW genes

The previous studies reported that the content of PAs gradually decreased during the development of strawberry fruits, while the opposite was true for anthocyanins, indicating that they were regulated by variable MBW complexes. So, the temporal and spatial expression profiles of *FaMYB1*/*5*/*9*/*10*/*11*, *FaEGL3*, *FaLWD1*, and *FaLWD1-like* were analyzed in the red-fleshed ‘Benihoppe’ and white-fleshed ‘Xiaobai’. *FaMYB1* and *FaMYB5* were constitutively expressed in different stages of developmental fruits of ‘Benihoppe’, but *FaMYB1* was gradually elevated in ‘Xiaobai’. *FaMYB10* was sharply induced in red fruits, especially in ‘Xiaobai’, while *FaMYB9* had relatively high expression levels in green fruits. The expression of *FaEGL3* was decreased with fruit ripening in ‘Benihoppe’ and ‘Xiaobai’. The transcription levels of *FaLWD1* and *FaLWD1-like* didn’t change much throughout the whole process of fruit development, but still lay particular stress on the late developmental stages (**Figure 5G**).

## Discussion

In our previous report (Jiang et al., 2023), it was preliminarily elucidated the interaction between FaMYB5, FaEGL3, FaLWD1 and FaLWD1-like proteins. Here, we expanded the test gene list to get the full landscape of the MBW complex in strawberry. Besides the TFs mentioned above, the new TFs are selected from the convincing data in the reported literature including 5 MYBs (FaMYB1, R3-FaMYB5, FaMYB9, FaMYB10, FaMYB11) 5 bHLHs (FabHLH33B, FabHLH33, FaGL3, FaMYC1, FabHLH3), and FaTTG1. FaMYB5 with an intact R2 domain is amplified, and R3-FaMYB5 is conjectured as a negative regulator of flavonoids biosynthesis (Schaart et al., 2013). We find both R3-FaMYB5 and R2R3-FaMYB5 could dimerize with themselves in different cell positions, R3-MYB5 protein is localized in the cell membrane, while R2R3-MYB5 is in the nucleus, and being in the nucleus is more conducive to its role as a TF (**Supplementary Figure S4**). What’s more, MYB related to anthocyanin metabolism is mostly R2R3 tape. All the above speculations indicate that this brand new R2R3-FaMYB5 may play an important role in regulating the accumulation of flavonoids in strawberry fruits. We indeed confirmed that R2R3-FaMYB5 promotes both anthocyanin and PAs accumulation, while Liu et al. (2022) demonstrated that *FaMYB5* is involved in citric acid metabolism. The MBW models reported that may regulate strawberry anthocyanin metabolism are: FaMYB10-FaEGL3 (FabHLH3/FabHLH33)-FaTTG1, and FaMYB1 might competitive combination with bHLH protein to balance anthocyanins and flavonoids accumulation (Schaart et al., 2013; Wei et al., 2018). However, we confirm that FaMYB10 does not interact with FabHLH33 by BiFC or Y2H, and Schaart also suggests that FabHLH33 has no interaction with the FaMYB1/5/9/11. Additional evidence for FabHLH33 might not be a member of the MBW complex is that FabHLH33 does not affect the anthocyanin pathway when knocked down using RNAi constructs (Lin-Wang et al., 2014). We clarify that the amino acid sequence of FabHLH33 is part of FaEGL3, and the bHLH TF involved in the regulation of flavonoids can bind to the DNA of MYB and WD40 through its very first 200 aa on the N-terminal side (Hichri et al., 2011), however, FabHLH33 lacks this part, this may be the reason why it cannot interact with MYB TFs. Another possible TF that may be in relation to the MBW complex is FaMYC1, for a feeble interaction could be detected between FaMYB5-FaMYC1, and FaMYC1-FaLWD1-like.

The ternary complex composed of FaLWD1 does not have the same intensity activation ability on structural genes like FaLWD1-like. The relative expression of *FaLWD1* is up-regulated by about 10-fold in *FaLWD1* OE, while *FaLWD1-like* dramatically reaches up to 20,000-fold in *FaLWD1-like* OE, and *FaLWD1-like* can be induced by positive regulators like FaMYB5 (**Supplementary Figures S3, 5**). The cell partition of FaLWD1-like is not only in the nucleus, but a large number of fluorescent spots could be observed in the cytoplasm (**Supplementary Figure S6**), such inducible expression properties and specific localization are not found in FaLWD1. The WD40 protein in the complex may act as a docking platform to transport the complex from the cytoplasm to the nucleus (Van Nocker and Ludwig, 2003; Hichri et al., 2011), so the incredibly high expression level and its specific localization of FaLWD1-like in strawberry fruits possibly accelerate the transport of the complex, thereby enhancing the regulatory ability of the complex. The MBW complex members do not include FaTTG1 for it does not interact with FaMYB1/5/10 in the BiFC strategy.

**Figure 6:**
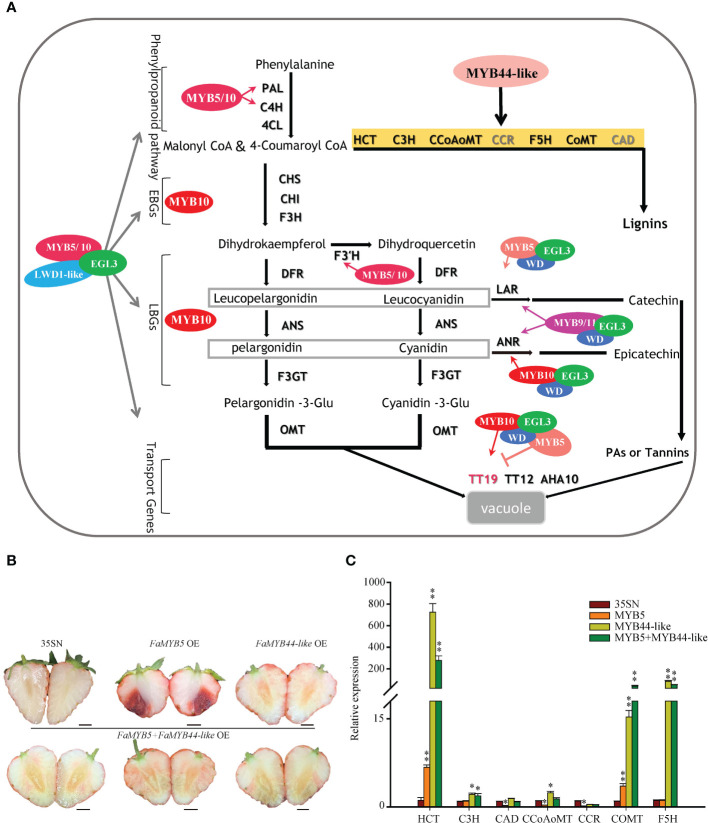
Regulation of flavonoids by MYBs and MBW complexes. **(A)** A proposed model for distinct regulation patterns of flavonoids by different MBW complexes in strawberry fruits. **(B)** The phenotype of co-overexpressed FaMYB5 and FaMYB44-like in ‘Xiaobai’. 35SN (empty vector) was as control, Scale bars represent 10 mm. **(C)** qRT-PCR analysis of relative expression levels of key structural genes of lignin metabolic pathway. Error bars are SEs for three replicates, statistical significance was determined by Student’s t-test (**, *P* < 0.01; *, *P* < 0.05).

In this study, we explore the model of MBW complexes dominated by FaMYB5 and FaMYB10 involved in the regulation of anthocyanins and PAs (**Figure 6A**): FaMYB5/10-FaEGL3-FaLWD1/FaLWD1-like. These two MYB TFs are found greatly promote the accumulation of anthocyanins and PAs in strawberry fruits. FaMYB10 boosts anthocyanins accumulation by increasing the expression of almost all structural genes in the metabolic pathway, while FaMYB10 does not specifically regulate the *LAR* of the PAs branch. PAs are synthesized respectively by two specific production pathways: *LAR* and *ANR*, and the increase of PAs in *FaMYB10* OE is mainly attributed to the accumulation of its upstream phenylpropane and flavonoids pathway metabolites, the *ANR* branch also contributes in part. Compared with FaMYB10, FaMYB5 is more specific, for the mRNA levels of *CHS*, *CHI*, *F3H*, *DFR2*, *ANS*, *F3GT*, *ANR*, and *TT19* have not been altered. However, *LAR* is significantly induced in *FaMYB5* OE, leading to a sharp increase in PAs. In addition, the two MYB TFs can restore the accumulation of Cy3G in strawberry flesh by raising the expression of *F3’H*. As far as the MBW ternary composed by FaMYB5 is concerned, the regulatory range on structural genes has become larger in *FaMYB5*-*FaEGL3* and *FaMYB5*-*FaEGL3*-*FaLWD1-like* OE. In addition to the above mentioned genes (*PAL*, *C4H*, *F3’H*, *LAR*, *TT12*, and *AHA10*) that are ascended in *FaMYB5* OE, *4CL2*, *CHS*, *CHI*, *F3H*, *ANS*, *F3GT* are also augment in the co-overexpressed samples, but *DFR2*, *ANR*, *TT19* still could not be affected by FaMYB5 or MBW complexes composed of FaMYB5. The *LAR* expression levels in *FaMYB10* OE or the complex that *FaMYB10* participates in are also invariably. Surprisingly, the promotion effect of the complex on structural genes is not much different or even weaker than that of FaMYB10 alone, maybe FaMYB10 play a too powerful role in the regulation of anthocyanins, weakening the effect of the complex, and the mixed agrobacterium liquid also dilutes the concentration of the *FaMYB10* overexpression vector to a certain extent.

In apple calluses, both *MdMYB9* and *MdMYB11* promote anthocyanins and PAs accumulation *via* binding to *ANS*, *ANR*, and *LAR* promoters (An et al., 2015). FaMYB9 and FaMYB11 are key MBW members involved in regulating PAs in strawberries (Schaart et al., 2013). We have proved that FaMYB9 and FaMYB11 restored the accumulation of Cy3G in ‘Benihoppe’ flesh by regulating the expression of *F3’H*, just like FaMYB5 and FaMYB10. The discrepancy remained between FaMYB9 and FaMYB11, FaMYB9 has a wider and deeper impact on the structural genes of the anthocyanin metabolic pathway, while FaMYB11 seems more finitude, only *F3’H* and *ANR* are upregulated more than 10 times. The different regulation model results in FaMYB9 regulates the total amount of anthocyanins by raising the content of Pg3G and restoring Cy3G, while FaMYB11 mainly by changing the ratio of Pg3G and Cy3G rather than increasing the total amount of anthocyanins. What’s more, both of them promote the accumulation of PAs through the key factors *LAR* and *ANR*, even more efficient than FaMYB5 and FaMYB10 in improving the production of PAs. In previous experiments we confirm that FaMYB9 and FaMYB11 interact with FaEGL3 and FaLWD1-like by the Y2H system, and reason that apart from FabHLH3 and FaTTG1, FaMYB9/11 may also function within MBW complex together with FaEGL3 and FaLWD1 or FaLWD1-like, mainly on the PAs branch. Comprehensively, considering the tissue-specific expression and the regulation mode of MYBs, it can be summarized that disparate MBW regulatory models fulfil a multitude of functions in different fruit development stages. FaMYB9 and FaMYB11 may responsible for the biosynthesis of flavonoids in the early fruit development stages for they are expressed in this period, while the late coloration of fruit is mainly controlled by FaMYB10. *FaMYB5* is stably expressed throughout the whole stages of fruit ripening and may play a complementary role in the biosynthesis of flavonoids.

Two types of R2R3-MYB that modulate anthocyanin biosynthesis, activators and repressors, repressors are classified into active-repressor and passive-repressor. Active-repressor possesses a conserved motif C2/EAR (pdLNLD/EL) in the C termini, while passive-repressor contains no repressive motif and both of them function by competing with R2R3-MYB activators for binding to a bHLH partner (Albert et al., 2014; Jun et al., 2015). Aharoni et al. (2001) demonstrated that FaMYB1 suppresses anthocyanin by adversely affecting the expression of late flavonoid biosynthesis genes enzyme activities. Later, Salvatierra et al. (2013) proved that the suppression of FcMYB1 triggered a transcriptional activation of *ANS*, leading to higher levels of Pg3G. All the evidence point out that FaMYB1 regulates the flavonoids pathway, including committed steps of anthocyanin biosynthesis and plays a role in the regulation of the branching point of the anthocyanin/PAs biosynthesis (LaFountain and Yuan, 2021). But does it work by competing with the activated MYB in combination with the bHLH cis-acting element? This is not yet known.

Given that FaMYB1 interacts with FaEGL3, the C-terminal of FaMYB1 includes a C2 motif, and we have affirmed that FaMYB1 inhibits the activation intensity of FaMYB5 on the *F3’H* and *LAR* promoters (**Figures 5B, C**). The spatiotemporal specificity analysis also showed that *FaMYB1* is mainly expressed at the late stage of fruit ripening (in this period the expression abundance of activated MYB, such as MYB10, is greatly increased), and the expression level of *FaMYB1* in ‘Xiaobai’ fruits is significantly higher than that in other stages and increases substantially when the fruits begin to turn red and reaches to the top in the full red stage. These clues suggest that FaMYB1 might negatively regulate anthocyanin metabolism through competitive binding to bHLH. To explore the intensity of FaMYB1 negatively regulating the accumulation of anthocyanins in strawberries, we choose ‘Benihoppe’ fruits with red pulp for the experiment. Overexpression of *FaMYB1* in ‘Benihoppe’ alone could not observe reduced pigmentation, and no significant phenotype could be found when it is co-injected with FaEGL3/FaLWD1/FaLWD1-like (**Supplementary Figure S7**). Hence we speculate that FaMYB1 is not the main factor that hinders the accumulation of anthocyanins in strawberries, it may fine-tune anthocyanin pigment intensity.

A cis-acting element TTTTTGCGGTTA (MYB binding site involved in flavonoids biosynthetic gene regulation) has come to light when analyzing the *FaMYB5* promoter by Plant Care. Online software http://planttfdb.gao-lab.org/ predicted that R3-type FaMYB44-like can bind to this element. Previous studies have proposed MYB44 competitively inhibits the formation of the MYB340-bHLH2-NAC56 complex to regulate anthocyanin biosynthesis in purple-fleshed sweet potato (Wei et al., 2020). Based on this, we believe that FaMYB44-like is likely to participate in the negative regulation of anthocyanin. To this end, the function of FaMYB44-like and FaMYB5 are verified in the ‘Xiaobai’ fruits and find that no red appears on the co-overexpressed flesh, the expression levels of *HCT* and *COMT* in the lignin synthesis pathway augment remarkably in the combination of *FaMYB5*+*FaMYB44-like* OE (**Figures 6B, C**). In-depth functional analysis of FaMYB44-like confirmed that it interferences the accumulation of anthocyanins by directing the metabolic flow of the phenylpropane pathway to the lignin tributaries. The internal molecular mechanism merits further exploration. In summary, the negative regulation of anthocyanins by MYBs mainly in the following three ways: (1) Negatively regulates the structural genes of the anthocyanin metabolic pathway directly;(2) Competing with transcriptional activators for bHLH to arrest the positive regulation of anthocyanin accumulation by the MBW complex; (3) Interfering with the flow of metabolism, directing to other branches such as phenolic acids or lignin.

## Data availability statement

The original contributions presented in the study are included in the article/**Supplementary Material**. Further inquiries can be directed to the corresponding authors.

## Author contributions

MY, LJ and HT designed the experiments. MY, NZ, YLiu and LZ performed the experiments. YLuo, YoZ and YW provided bioinformatic support. YLin and YuZ analyzed the data. MY wrote original draft. ML, XW, QC and HT wrote and edited.
