# Supplementary material for: Regulation of flavonoids in strawberry fruits by FaMYB5/FaMYB10 dominated MYB-bHLH-WD40 ternary complexes
Source: Front Plant Sci. 2023 Mar 13;14:1145670. doi: 10.3389/fpls.2023.1145670 (PMC10040760; doi:10.3389/fpls.2023.1145670)
Supplement: Supplementary file 1 [file DataSheet_1.docx]

**Regulation of strawberry fruit flavonoids by multiple MYB-bHLH-WD40 ternary complexes**

**Maolan Yue ^1†^, Leiyu Jiang ^1†^, Nating Zhang ^1^, Lianxi Zhang ^1^, Yongqiang Liu^1^, Yuanxiu Lin^1,2^, Yunting Zhang^1,2^, Ya Luo^1^, Yong Zhang^1^, Yan Wang^1,2^, Mengyao Li^1^, Xiaorong Wang^1,2^, Qing Chen^1^*, Haoru Tang^1,2^***

^1^Country College of Horticulture, Sichuan Agricultural University, Chengdu, China

^2^ Institute of Pomology & Olericulture, Sichuan Agricultural University, Chengdu, China

*** Correspondence:**Qing Chen, supnovel@sicau.edu.cn
Haoru Tang, htang@sicau.edu.cn

**Supplementary Figures list**

**Figure S1** The phenotype of transient overexpression MYBs in ‘Benihoppe’ fruits.

**Figure S2** Analysis of Cy3G and Pg3G by HPLC method.

**Figure S3** Relative expression levels of genes in their own overexpressed samples.

**Figure S4** R3-FaMYB5 and R2R3-FaMYB5 dimerized in different cell regions.

**Figure S5** Relative expression levels of *FaLWD1* and *FaLWD1-like* in *FaMYB5* OE.

**Figure S6** FaLWD1 and FaLWD1-like dimerized in different cell regions.

**Figure S7** The phenotype of transient overexpression FaMYB1, FaEGL3 and FaLWD1-like in ‘Benihoppe’ fruits.


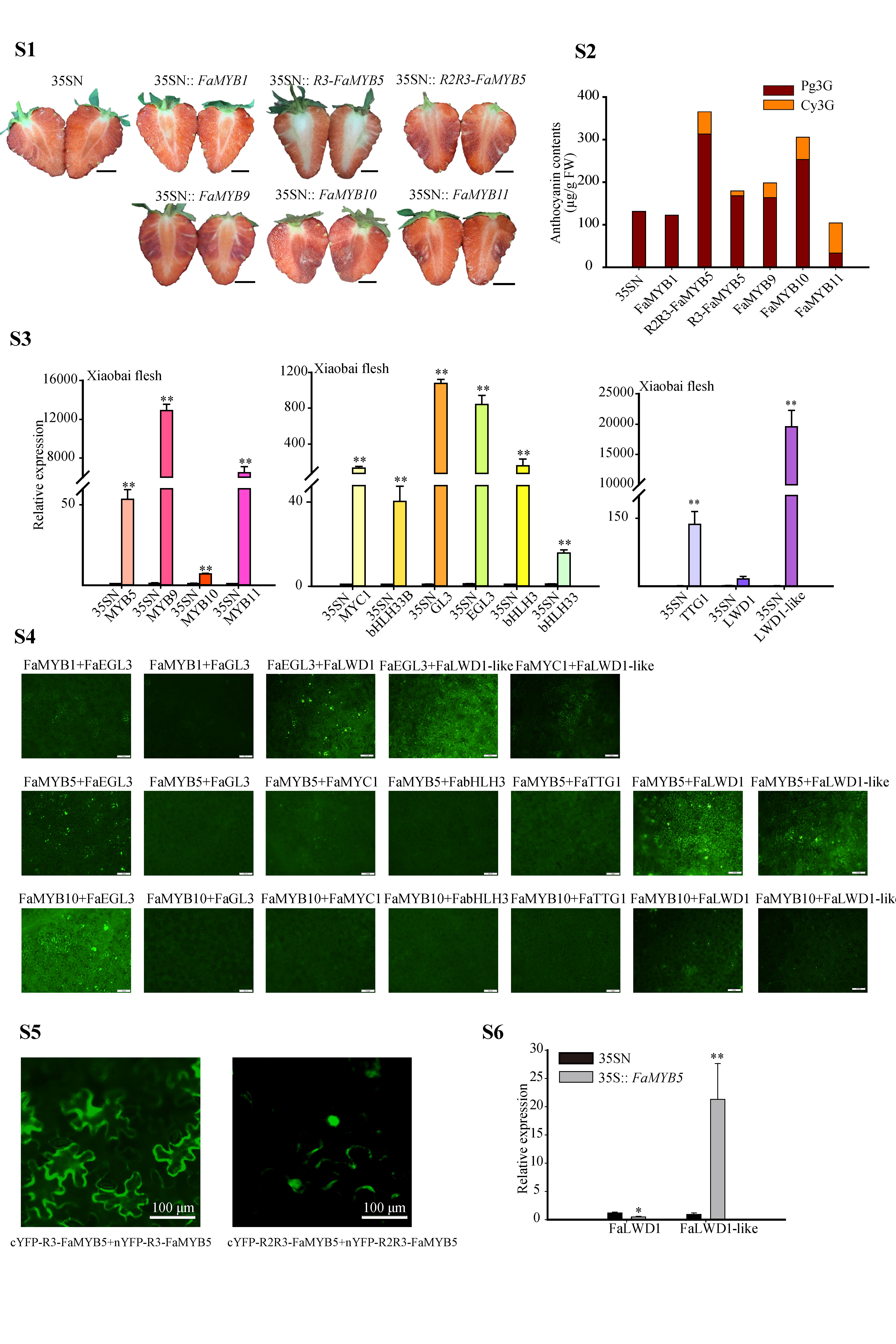


**Figure S1** The phenotype of transient overexpression MYBs in ‘Benihoppe’ fruits.


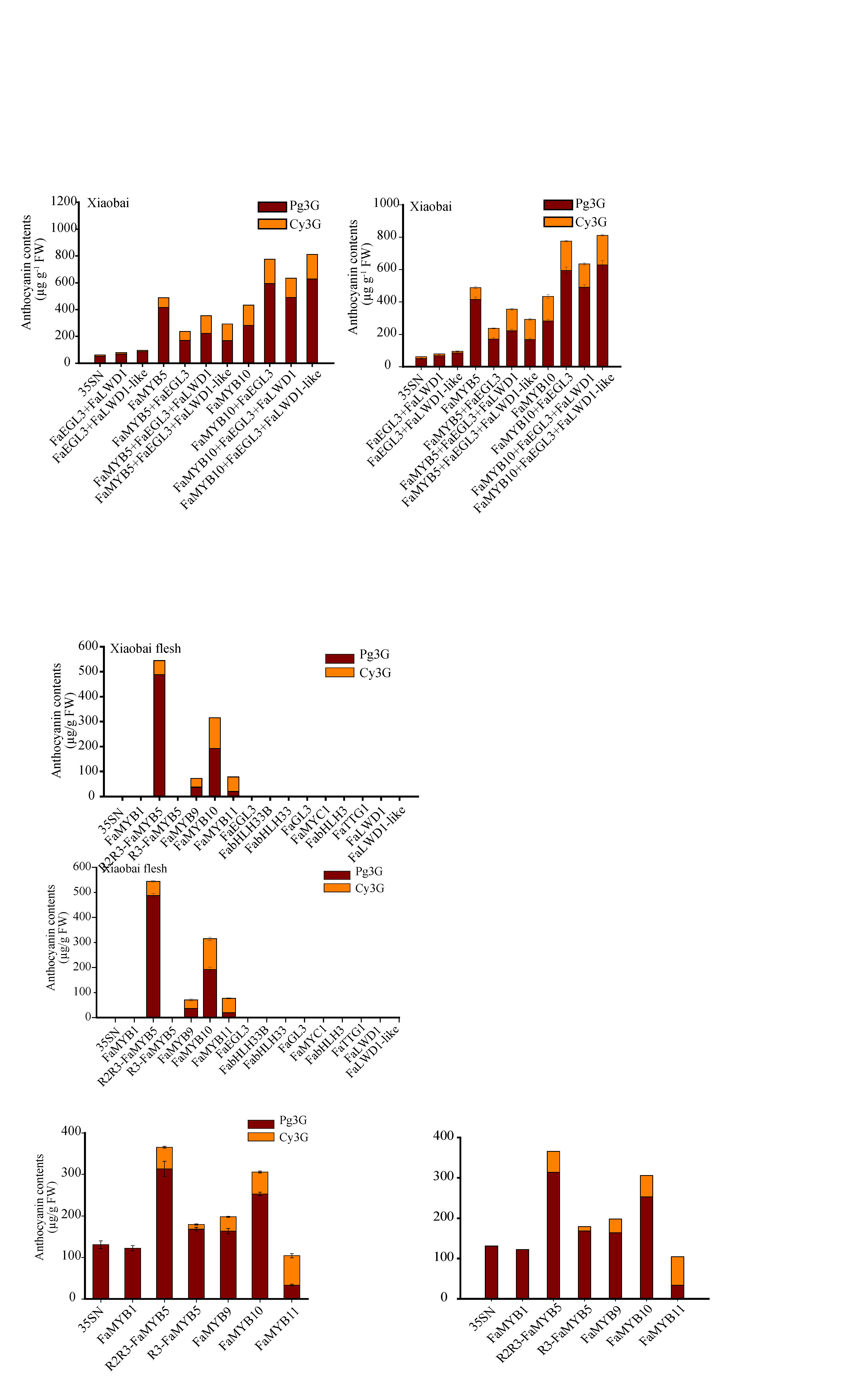


**Figure S2** Analysis of Cy3G and Pg3G by HPLC method.


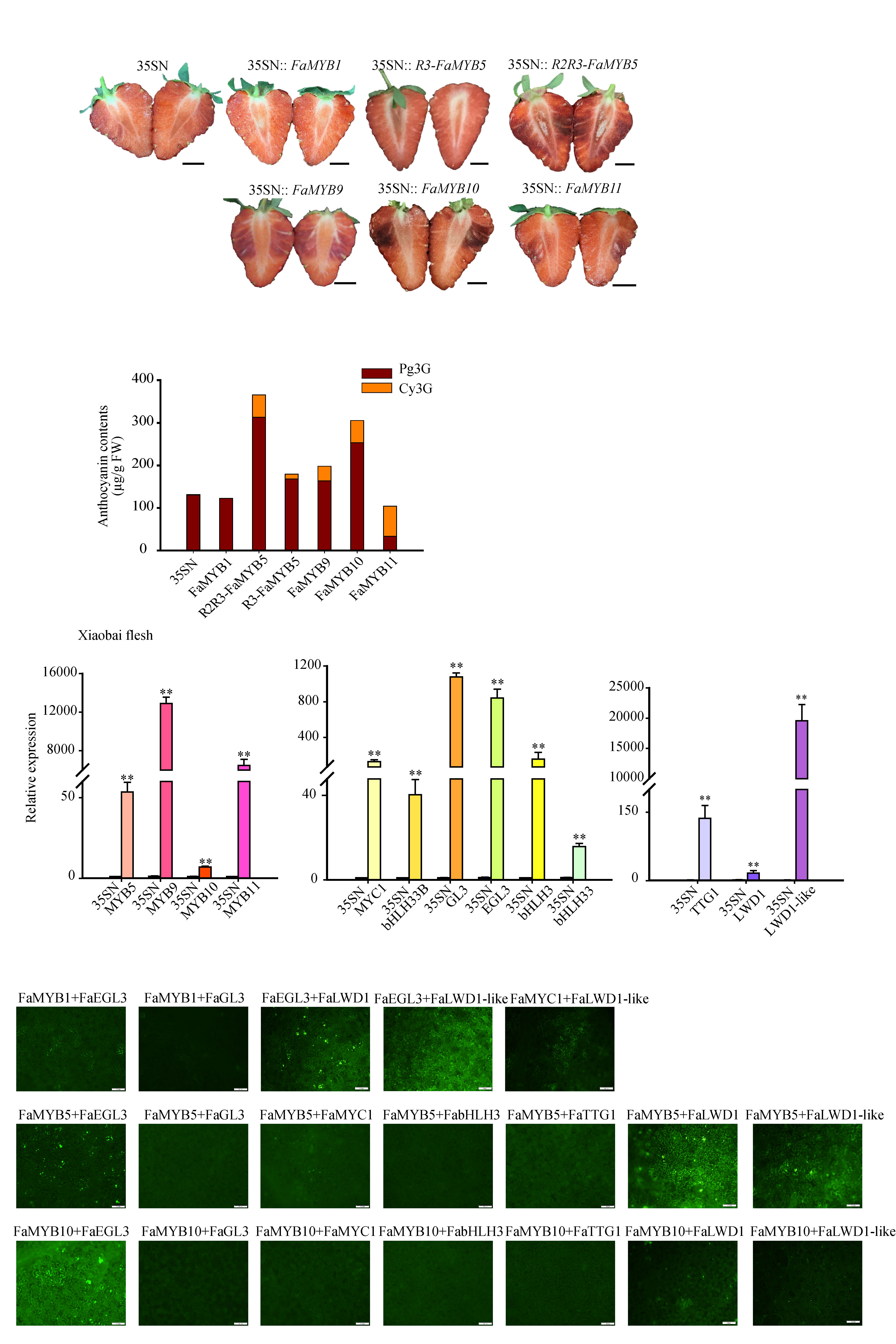


**Figure S3** Relative expression levels of genes in their own overexpressed samples.


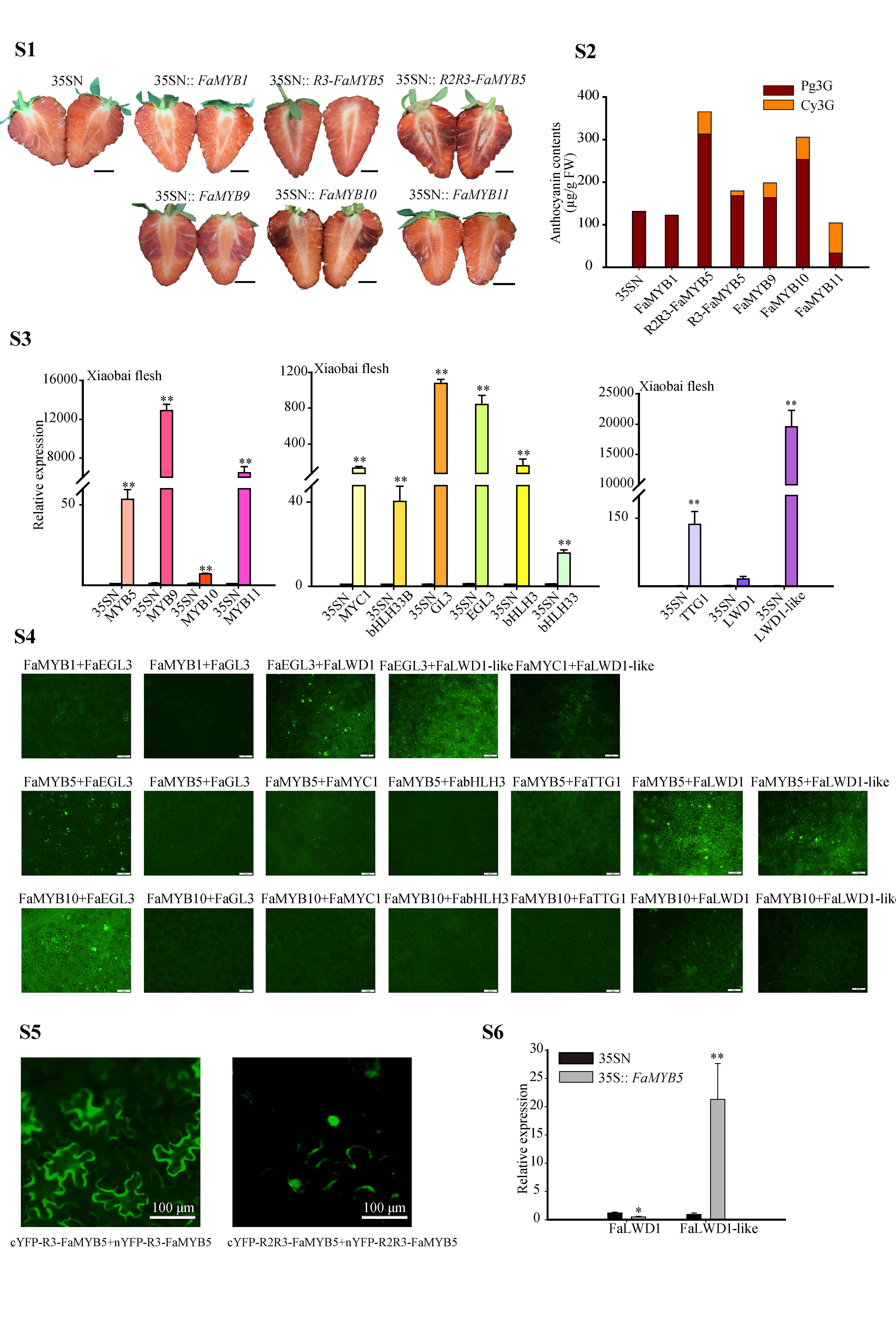


**Figure S4** R3-FaMYB5 and R2R3-FaMYB5 dimerized in different cell regions.


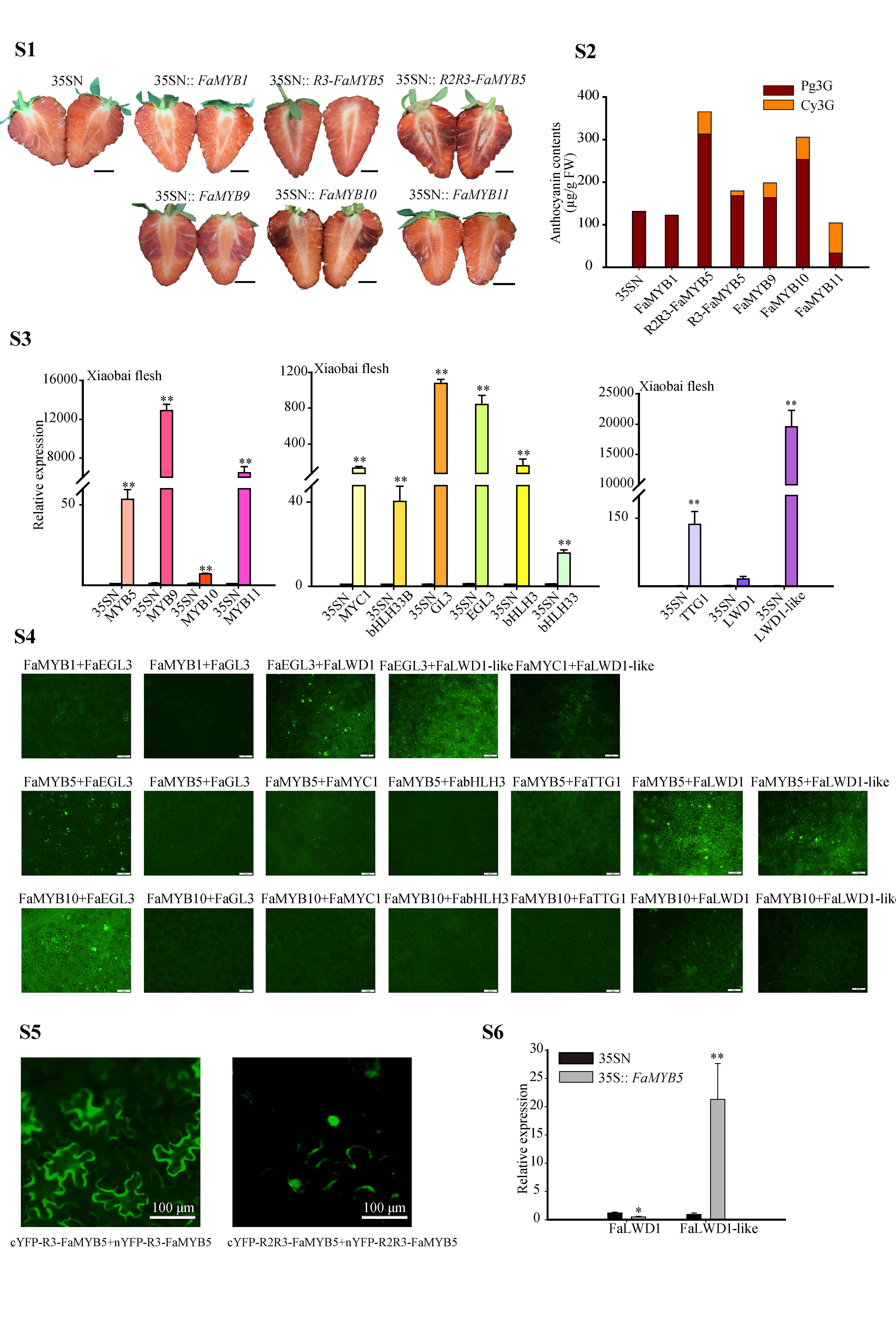


**Figure S5** Relative expression levels of *FaLWD1* and *FaLWD1-like* in *FaMYB5* OE.


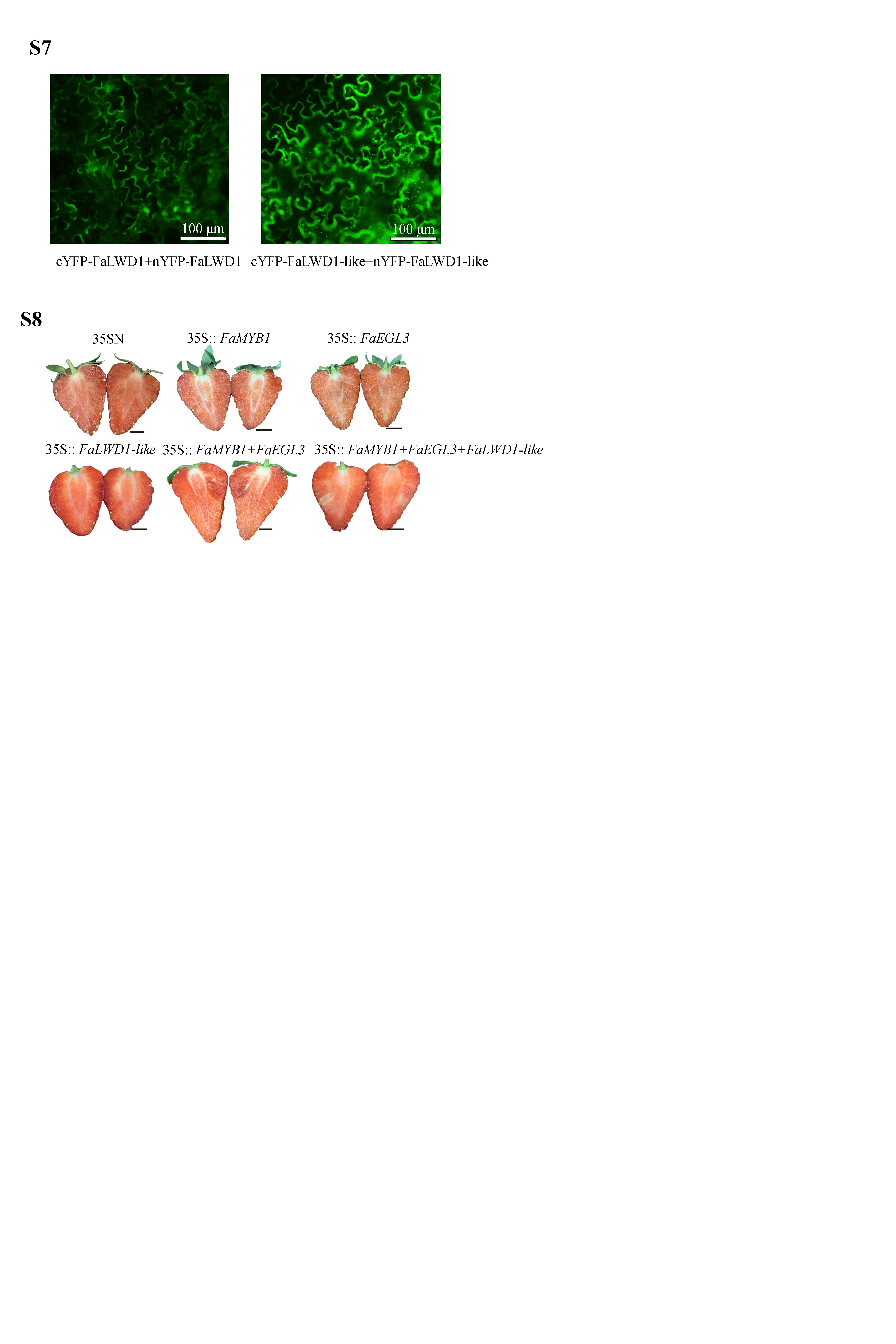


**Figure S6** FaLWD1 and FaLWD1-like dimerized in different cell regions.


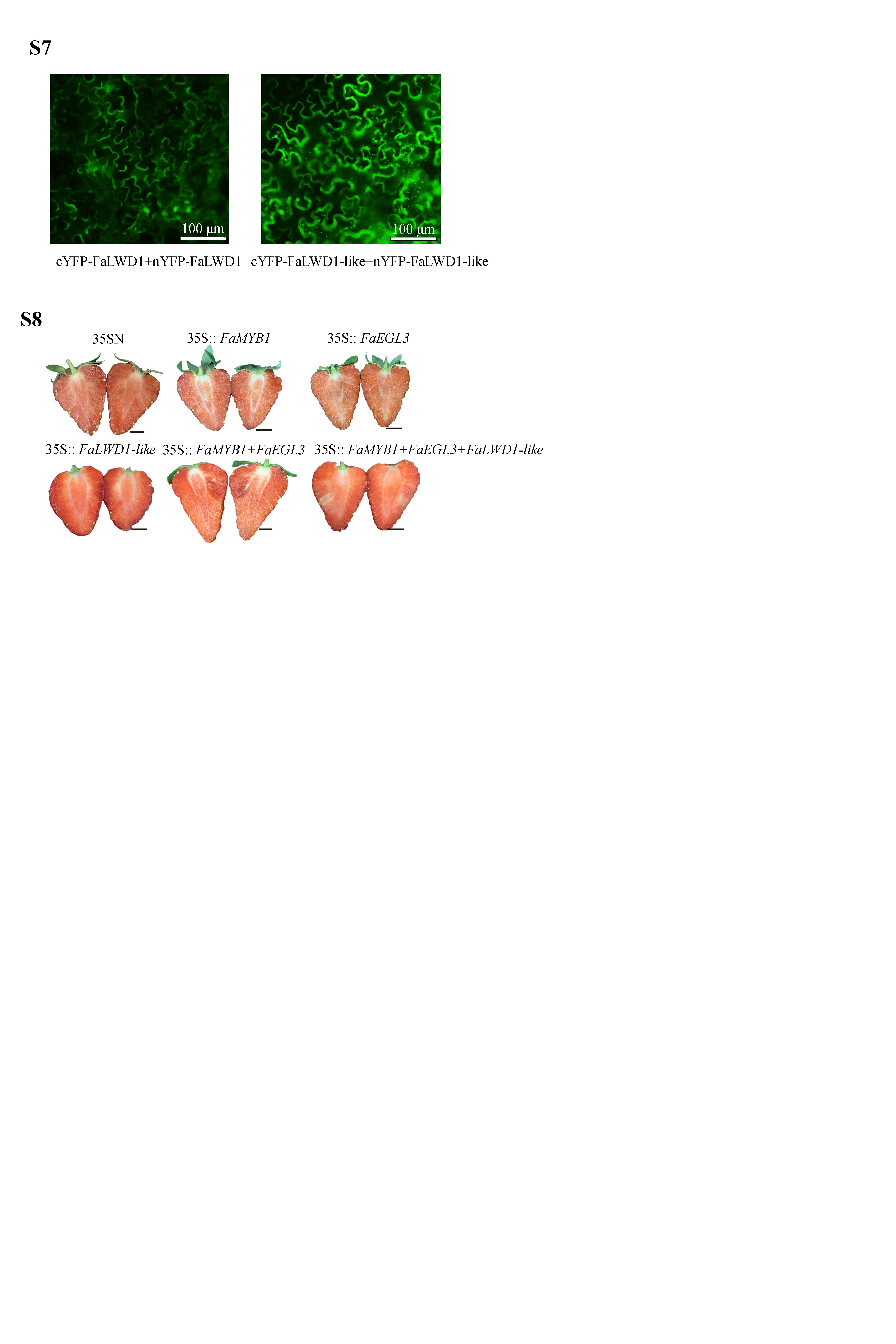


**Figure S7** The phenotype of transient overexpression *FaMYB1*, *FaEGL3* and *FaLWD1-like* in ‘Benihoppe’ fruits.
